# An (Un)Holy Trinity: Differences in Climate Change-Induced Distress Between Believers and Non-believers in God Disappear After Controlling for Left–Right Political Orientation

**DOI:** 10.1007/s10943-022-01706-2

**Published:** 2022-12-01

**Authors:** John B. Nezlek, Marzena Cypryańska

**Affiliations:** 1 Center for Climate Action and Social Transformations, Institute of Psychology, SWPS University of Social Sciences and Humanities, ul. Chodakowska 19/31, 03-815 Warsaw, Poland; 2 Department of Psychological Sciences, College of William & Mary, Box 8795, Williamsburg, VA USA

**Keywords:** Belief in God, Religiosity, Climate change, Climate change distress, Well-being, Political orientation

## Abstract

We examined differences in reactions to climate change as a function of belief in God. We studied four samples, convenience samples of university students in the USA (*n* = 627) and in Poland (*n* = 628), a nationally representative sample of adults in Poland (*n* = 1154), and a nationally representative sample of adults in the USA (*n* = 1098). In each study we measured the distress people felt about climate change, belief in God, and left–right political orientation. These constructs were measured slightly differently across the studies. Regardless of how these constructs were measured, believers were less distressed by climate change than non-believers, and with only a few exceptions, these differences disappeared after covarying political orientation (left–right or liberal-conservative). Contrary to those who argue that there is something inherent in religious belief that predisposes people to deny or ignore climate change, the present results suggest that it is the (growing) confluence of faith and conservative political orientation that is responsible for the fact that some people of faith tend to deny climate change or actively oppose efforts to combat it.

## Introduction

The present set of studies examined differences in reactions to climate change as a function of people’s belief in God. More specifically, we believed that such differences would reflect individual differences in left–right political orientation and that when individual differences in political orientation were controlled, we expected that differences in reactions to climate change as a function of belief in God would decrease or disappear. This expectation was based on the results of three overlapping bodies of research: research about relationships between political orientation and beliefs about climate change, research about relationship between political orientation and religious belief, and research about relationships between religiosity and beliefs about climate change. We focused on left–right political orientation because it is the most widely studied measure of political orientation. We focused on climate change-induced distress because such distress can prompt people to take action to mitigate climate change (Verplanken & Roy, 2013; Verplanken et al., 2020), a possibility we discuss below.

Below, we briefly review research on these three topics. These reviews are not comprehensive. Each of these three topics has been the subject of numerous studies, and comprehensive reviews were not needed (or practical) for present purposes. We have presented research that represents the gist of research on these topics. Following this, we describe the present study and our expectations regarding the results.

### Political Orientation and Beliefs About Climate Change

The present studies were specifically concerned with the roles left–right political orientation plays in relationships between religiosity and reactions to climate change. An important context for this research is the well-documented negative relationship between conservative political beliefs and the recognition of global warming (Dunlap & McCright, 2008). As noted by Hornsey et al. (2016) in their meta-analysis of 171 studies across 56 nations: “People who intend to vote for more liberal political parties are more likely to believe in climate change than those who align themselves with relatively conservative political parties… its effect is roughly double the size of any other demographic variable.”

Much of this research has been done in the USA (e.g., 48% of the studies cited by Hornsey et al.), and some have suggested that this “political divide” does not exist in all countries, e.g., former Communist countries in Europe in which climate change is not salient (McCright et al., 2016). Although there may be country-level differences in the strength of this divide, McCright et al. analyzed data that were collected in 2008, and more recent data suggest that their specific conclusion may need to be revisited. For example, Czyżewski et al. (2022) analyzed data collected in Poland in 2017 and found a negative relationship between conservative political orientation and support for pro-environmental activities. Moreover, although Hornsey et al. found differences between US and non-US samples in some relationships, they did not find that these relationships did not exist in both US and non-US samples; they simply varied in strength.

NB: In the US context, political orientation is typically discussed and measured in terms of liberal versus conservative, corresponding quite closely to the left–right distinction that is used in much of the rest of the world. In Poland, as in some other European countries, the liberal-conservative distinction does not map cleanly onto the left–right distinction. So, in our US studies we measured liberal-conservative, and in our Polish studies, we measured left–right. Consistent with how this distinction is discussed in much of the research on this topic, we refer to liberal-conservative, with occasional uses of left–right to indicate that our results hold for both sets of terms.

### The Relationship Between Political Orientation and Religious Belief: The Confluence of Politics and Faith

The next “piece of the puzzle” is the relationship between political orientation and religious belief. Although there are differences in this relationship as a function of how each construct is measured, generally, there is a positive relationship between religiosity and political conservatism. For example, in a 2014 study of 35,000 Americans, PEW Research found that 85% of conservatives identified as Evangelical Protestants (Pew Research Center, 2015), although Jews, Muslims, Buddhists, and Hindus tended to be more liberal than Christians. Based on analyses of the World Values Survey (pooled 1981–2001) it appears that similar differences exist worldwide (Norris & Inglehart, 2011, pp. 201–208). Although Norris and Inglehart suggest that religiosity and the strength of its relationship with political orientation have declined somewhat over the past 30 years, there are reasons to suspect that this relationship may have stabilized or may be getting stronger.

For example, in a chapter entitled “The next Middle Ages: religion and political culture” Graziano (2020, pp. 15–18) noted: “Islam and Christianity—but also Buddhism, Hinduism, Judaism, etc.—provide a more secure and cohesive foothold of collective identity than the state: in fact, in the collective imagination, they represent a mythological golden age when populations identified within themselves a common religious feeling which served both as a banner and as a protective shield.” He continued: “To conclude, it remains to be seen on what level the current relationship between religion and politics is situated at a time when religion increasingly neglects the spiritual realm and when politics in an increasingly evident way takes on the appearance of religious beliefs.” He summed up with: “After all, adds Roy, ‘religion does not require people to know, but to believe’ (Roy, 2013: 6). Today, when politics too ‘does not require people to know, but to believe’, we witness a new reconciliation between the two camps. It is a soft return to the Middle Ages, in which both politics and religion lose their specific features and are drawn toward the flattening of an ignorance which, although it still preserves its formal pretense, has nothing ‘holy’ anymore.”

### Religiosity and Climate Change

The upshot of research on religiosity and climate change is that religiosity is negatively related to beliefs that climate change is due to human activity, to environmental concern, to support for environmental policies, and so forth. The classic reference for this is White (1967). Although this citation is over 50 years old and some have argued that the relationships White described do not hold as strongly now as they did then (e.g., Wilkinson, 2010), it appears that the situation has not changed much in the past 50+ years (Konisky, 2018; Taylor et al., 2016). A similar conclusion was reached by Arbuckle (2017, p. 181).

There have been attempts within communities of faith to reverse this tendency. For example, within the Christian tradition, some have argued for a stewardship perspective (Kearns, 1996; Shaiko, 1987), an emphasis on the world as God’s gift to humanity with the understanding that humanity is responsible for caring for this gift. Unfortunately, in terms of enlisting Christians in the fight against climate change, Arbuckle (2017) concluded that there was “little evidence that stewardship beliefs hold prominence in Christian religious traditions (p. 4).” The difficulty in overcoming the negative relationship between religiosity and pro-environmental attitudes is illustrated by the impact on Catholics in the US of Pope Francis’s, 2015 encyclical Laudato Si (Pope Francis, 2015). As noted by Li et al. (2016). “Cross-pressured by the inconsistency between the pontiff’s views and those of their political allies, conservative Catholics devalued the Pope’s credibility on climate change.” Apparently, to conservative Catholics in the US, the Pope is not infallible.

### Climate Change Distress

There is broad agreement that climate change is reducing people’s mental and physical health worldwide (e.g., Clayton et al., 2017). In terms of mental health, although much of the research and theorizing has focused on anxiety (e.g., Clayton, 2020), leading to the use of the phrase “eco-anxiety,” research has found that people who are aware of the negative consequences of climate change can experience a broad range of negative emotions such as anxiety, fear, worry, frustration, anger, or sadness, and grief (Cunsolo & Ellis, 2018; Searle & Gow, 2010). For reviews see Ojala et al. (2021) and Pihkala (2020). Given this, we measured various negative affective states, and we refer to these measures collectively as climate change-induced distress.

Moreover, it is important to distinguish climate change-induced distress from psychological disorder. For example, in a classic paper, Horwitz (2007, p. 274) distinguished “the psychological distress that non-disordered people naturally develop under stressful circumstances from mental disorders.” Similarly, in terms of climate change, Clayton (2020) noted that “it is important to distinguish between adaptive and maladaptive levels of anxiety in terms of climate change.”

We conceptualized climate change-induced distress as a natural emotional reaction to threat and loss. As Verplanken and Roy (2013) noted, experiencing distress due to thinking about climate change is perfectly normal. Climate change is a threat to the world, and people should feel distress when they think about it. Moreover, distress is arousing, and as such, it can serve as a motivator for action. Such possibilities figure prominently in models of coping such as Witte’s Extended Process model (Maloney et al., 2011) and Lazarus and Folkman’s model of stress and coping (Folkman & Lazarus, 1988).

The type of non-pathological distress we are considering here has been found to be adaptive in terms of reacting to threats (Cypryańska & Nezlek, 2020), and in terms of motivating people to take action to combat climate change (Verplanken & Roy, 2013; Verplanken et al., 2020). As noted by Brosch (2021) in his review: “The affective responses people experience toward climate change are consistently found to be among the strongest predictors of risk perceptions, mitigation behavior, adaptation behavior, policy support, and technology acceptance (p. 15).”

Although climate change-induced distress and its variants have figured prominently in recent research on climate change, we are unaware of research that has examined relationships between distress and religiosity, and we are aware of only one study that has examined relationships between distress (defined as solely in terms of worry) and political orientation (Gregersen et al., 2020). This study found a negative relationship between worrying about climate change and left–right orientation.

### Conceptualizing Religiosity

Religiosity has been conceptualized and measured in myriad ways (e.g., Hill & Maltby, 2009; Koenig et al., 2015). Although there are good reasons to conceptualize religiosity in different ways, for present purposes, we used a simple measure: belief in God. As noted by Nezlek (2022, p. 2573) “Belief in God is a foundation of religious faith, and various measures of religiosity are manifestations of this fundamental belief.” Moreover, using a dichotomous measure of belief in God, Nezlek found that “that people who believed in God were less ideologically prosocial and were less civically involved than those who do not believe in God (p. 2582).” Such differences are consistent with our contention that religiosity is negatively related to concerns about climate change. We address the issue of conceptualizing religiosity in studies of climate change in the discussion.

### The Present Study

We believe that the existing research has not examined the mutual relationships among climate change-induced distress, religiosity, and political orientation. Research has examined mutual relationships among political orientation, religiosity, and perceptions of climate change (Arbuckle, 2017; Li et al., 2016). Although valuable, the existing research has not examined climate change-related distress. To fill this gap, we analyzed the data from four studies. Our guiding hypothesis was that differences in climate distress between those who believed in God and those who did not would not exist or be diminished after controlling for left–right political orientation. We also controlled for socio-demographic characteristics for individual differences in sex, age, education, and ethnicity, demographic characteristics that are frequently taken into account in survey research. This was done to ensure that whatever effects we found for political orientation could not also be explained by individual differences in demographic characteristics.

### Background for Studies

All studies were approved by an IRB of one of the authors’ home institutions. All participants in all studies provided written (electronic) consent. Participants in Studies 1, 2, and 4 were given an “I chose not to respond” option (or a functional equivalent) for all questions. Participants in Study 3 or were allowed to terminate participation at any time without penalty. The data for all studies are available via the Open Science Foundation repository (https://doi.org/10.17605/OSF.IO/2H6ER. The repository contains, separately for each study, a SPSS data file, a codebook, and a csv file containing the data.

## Study 1

### Methods: Sample and Measures

Participants in Study 1 were 627 undergraduates who were attending a university in the United States (62% women, *M*_age_ = 18.8; SD = 1.47) and participated in the study as partial fulfillment of a course requirement. Of these 627, 616 answered a question about their belief in God: “Do you believe in God?” using a response scale with options “no,” “not certain,” and “yes.” Of these 616, 129 (21%) answered no, 213 (35%) answered not certain, and 274 (44%) answered yes. Of the total sample of 627, 554 described their religious affiliation: 270 were Christian, 18 were Jewish, 14 were Muslim, 19 were Hindu or Buddhist, 98 were atheists, and 135 were other. In terms of ethnicity, 74% of the sample identified as White, 16% as Asian, and 6% as Black/Africa/American. The study was conducted from August to December of 2021.

Participants described their beliefs about climate change by responding to the question: “Which of the following statements best describes your beliefs about climate change?” The response options (and percent endorsing each response) were: “I do not think the climate is changing” (0.3%); “It is not certain if the climate is changing” (1.8%); “I think that climate change is happening, but it is entirely caused by natural processes” (1.1%); “I think that climate change is happening, but it is mainly caused by natural processes” (2.2%); “I think that climate change is happening, and it is due to an equal mix of natural processes and human activity” (15.3%); “I think that climate change is happening, and it is mainly caused by human activity” (60.1%); and “I think that climate change is happening, and it is entirely caused by human activity” (19%).

It is important to note that according to the most recent report of the Intergovernmental Panel on Climate Change (IPCC; Pörtner et al., 2022), the only truly correct answer is the last response (climate change is human caused), although for present purposes, we have classified the next to last response (climate change is mainly caused by human activity) as correct. All other responses are clear indications of climate change denialism. In light of this, we created a variable, denialism, coded 1 for participants who endorsed options 1 through 5 on the measure of beliefs about climate change (21%) and coded 0 for participants who endorsed options 6 or 7 on the measure of beliefs about climate change (79%).

Climate change-induced distress was measured with four items, and 627 participants responded using 5-point scales labeled: (1) I do not feel this way at all; (2) I feel this way slightly; (3) I feel this way moderately; (4) I feel this way strongly; and (5) This is exactly how I feel. The items, summary statistics, and frequency counts for these measures are presented in Table 1. As can be seen from these data, mean responses were above the midpoint of the scale, and all the distributions of all responses were negatively skewed. Approximately 50% of respondents endorsed either strongly or exactly how I feel.

**Table 1:** Study 1: Descriptive statistics and of responses for measures of climate change distress. Percent for each response in parentheses

Item			Response				
	*M*	SD	1	2	3	4	5
I fear the consequences of climate change	3.68	1.29	46 (7.3)	85 (13.6)	117 (18.7)	153 (24.4)	226 (36.0)
I worry about what the future will be like because of climate change	3.68	1.32	50 (8.0)	85 (13.6)	121 (19.3)	133 (21.2)	238 (38.0)
Climate change makes me feel anxious	3.20	1.41	97 (15.5)	117 (18.7)	141 (22.5)	108 (17.2)	163 (26.0)
I am concerned about climate change	3.73	1.26	40 (6.4)	77 (12.3)	133 (21.2)	141 (22.5)	236 (37.6)

Political orientation was measured with a single item: Please describe your general political orientation using the following scale (with percent endorsing each option of the 596 participants who answered this question): (1) Very liberal (16%), (2) Liberal (32%), (3) Slightly liberal (19%), (4) Moderate, middle of the road (18%). (5) Slightly conservative (8%), (6) Conservative (6%), and (7) Very conservative (2%). Recall that this dimension corresponds to the left–right dimension measured in the Polish studies.

### Results

#### Verifying Assumptions

The present hypotheses were predicated on previous research showing that:

1. Religiosity was positively related to endorsing conservative political ideologies,
2. Religiosity was negatively related to believing the truth about climate change, and
3. Endorsing conservative political ideologies was negatively related to believing the truth about climate change.

The first assumption was confirmed by the results of a one-way ANOVA in which belief in God was the independent variable and political orientation was the outcome, *F*(2, 584) = 51.2, *p* < 0.0001. As expected, believers were more conservative (*M* = 3.56) than both non-believers (*M* = 2.41), and those who were uncertain (*M* = 2.40) (ps < 0.0001 for both comparisons using Scheffe test).

The second assumption was confirmed by the results of chi-squared analysis that crossed belief in God with denialism, *χ*^2^(2) = 64.3, *p* < 0.0001. Believers were more likely to deny that climate change was human caused (35%) than either non-believers (8%) or those who were uncertain (10%).

The third assumption was confirmed by the results of a *t*-test in which denialism was the independent variable and political orientation was the outcome, *t*(594) = 14.2, *p* < 0.0001. Climate change deniers were more conservative, *M* = 4.41, than accepters of the truth, *M* = 2.54.

#### Climate Change-Induced Distress and Belief in God

Next, we examined differences in climate change-induced distress as a function of belief in God. These analyses included the 587 participants who answered the belief in God, political orientation, and climate change distress questions.

The first analysis was a one-way ANOVA in which belief in God was the independent variable and the four measures of climate change-induced distress were the dependent variables. The results of these analyses are summarized in Table 2. These analyses found a significant main effect for belief in God for all four outcomes, and as can be seen from the means presented in Table 2, those who believed in God were less distressed by climate change than non-believers or those who were uncertain. Contrasts of believers versus non-believers + uncertain were all significant at *p* < 0.00001.

**Table 2:** Study 1: Climate change-induced distress as a function of belief in God

Outcome	Unadjusted means				Means with political orientation as a covariate				Means with political orientation, sex, age, and ethnicity as covariates			
	Do not believe	Not certain	Believe	*F*	Do not believe	Not certain	Believe	*F*	Do not believe	Not certain	Believe	*F*
Fear consequences	4.01	3.98	3.33	20.52	3.78	3.75	3.62	1.07	3.81	3.73	3.66	< 1
Worry about future	3.90	4.03	3.35	18.35	3.67	3.80	3.64	1.15	3.70	3.78	3.66	< 1
Feel anxious	3.51	3.52	2.87	15.98	3.25	3.26	3.21	< 1	3.25	3.24	3.24	< 1
Concerned about climate change	4.02	4.09	3.39	22.28	3.80	3.87	3.67	1.83	3.82	3.85	3.71	< 1

*F* ratio is the effect for belief in God. All main effects of unadjusted means were significant at *p* < .00001. *n*s = 587, 587, 586 and 587, respectively

The next analysis was a one-way ANCOVA in which belief in God was the independent variable, the four measures of climate change-induced distress were the dependent variables, and political orientation was a covariate. The results of these analyses are summarized in Table 2. As expected, after controlling for political orientation, all differences in distress as a function of belief in God were rendered not significant (all ps > 0.15). The adjusted means are presented in Table 2.

The next analyses were ANCOVAs with political orientation, sex, age, and ethnicity as covariates. The sample was 75% self-described as White, and ethnicity was represented with a dummy-coded variable, 0 = not White, 1 = White. Age was measured in self-reported years. Gender was represented with a dummy-coded variable, 0 = male, 1 = female. This was a student sample, and so education was not included as a covariate. The results of these analyses are presented in Table 2. As can be seen from the adjusted means in this table, the inclusion of sex, age, and ethnicity did not alter the results meaningfully. Differences between the two sets of adjusted means were in the second decimal place.

## Study 2

### Methods: Sample and Measures

Participants in Study 2 were 628 undergraduates who were attending a university in Poland (83% women, *M*_age_ = 26.9; SD = 8.20) and participated in the study as partial fulfillment of a course requirement. Of these 628, 558 answered a question about their belief in God: “Do you believe in God?” using a response scale with options “no” and “yes.” Of these 558, 270 (48%) answered no, and 288 (52%) answered yes. Of the total sample of 628, 521 described their religious affiliation: 296 were Christian (86% Catholic), 6 were Buddhist, and 199 were other. Given the centrality of belief in God to the present study, unless otherwise noted, all analyses described below were based on the sample of 558 who answered the belief in God question. The study was conducted in March and April of 2021. Poland is 97% ethnically Polish (The World Factbook, 2021), and so ethnicity was not measured in this study.

Participants described their beliefs about climate change by responding to the same question used in Study 1.The percent endorsing each response were: “I do not think the climate is changing” (0.2%); “It is not certain if the climate is changing” (0.5%); “I think that climate change is happening, but it is entirely caused by natural processes” (1.1%); “I think that climate change is happening, but it is mainly caused by natural processes” (2.5%); “I think that climate change is happening, and it is due to an equal mix of natural processes and human activity” (17.6%); “I think that climate change is happening, and it is mainly caused by human activity” (70.4%); and “I think that climate change is happening, and it is entirely caused by human activity” (7.6%). Similar to study 1, a measure of climate change denial was created (endorsing options 1 through 5), and similar to study 1, 23% of participants did not acknowledge the reality of climate change.

Climate change-induced distress was measured with seven items. Each was preceded by the stem “How often do you feel…”, and response options were: (1) Not at all, (2) Very rarely, (3) Rarely, (4) Occasionally, (5) Often, (6) Very often, and (7) All the time. The items, summary statistics, and frequency counts for these measures are presented in Table 3. Mean responses tended to be just below the scale midpoint (except for the item about joy of life). Unlike Study 1, the distributions of most of the items were not characterized by any pronounced skew.

**Table 3:** Study 2: Descriptive statistics and of responses for measures of climate change distress. Percent for each response in parentheses

Item			Response						
	*M*	SD	1	2	3	4	5	6	7
Due to climate change, I am worried about what the future will be like	3.82	1.52	53 9.5)	67 (12.0)	73 (13.1)	173 (31.0)	116 (20.8)	60 (10.8)	11 (2.0)
I feel anxious about the progressive climate change	3.68	1.55	68 (12.2)	63 (11.3)	88 (15.8)	175 (31.4)	91 (16.3)	58 (10.4)	12 (2.2)
I am afraid that the effects of climate change will be fatal	3.97	1.53	45 (8.1)	68 (12.2)	62 (11.1)	167 (29.9)	122 (21.9)	74 (13.3)	16 (2.9)
I feel dread when I think about the consequences of climate change	3.56	1.64	87 (15.6)	68 (12.2)	91 (16.3)	150 (26.9)	85 (15.2)	61 (10.9)	13 (2.3)
I feel sad when I think about the progressive climate change	3.92	1.55	48 (8.6)	70 (12.5)	68 (12.2)	165 (29.6)	112 (20.1)	78 (14.0)	14 (2.5)
The awareness of the possible consequences of climate change takes away the joy of life	2.72	1.50	162 (29.0)	102 (18.3)	119 (21.3)	107 (19.2)	35 (6.3)	26 (4.7)	4 (.7)
The future seems hopeless to me when I think about climate change	3.40	1.63	97 (17.4)	80 (14.3)	97 (17.4)	132 (23.7)	91 (16.3)	43 (7.7)	13 (2.3)
I feel sorry for the consequences of climate change	4.18	1.52	39 (7.0)	52 (9.3)	59 (10.6)	151 (27.1)	151 (27.1)	81 (14.5)	23 (4.1)

Political orientation was measured with a single item: Please describe your general political orientation using the following scale, with endpoints labeled (1) definitely left and (10) definitely right. Just over half (55%) of the 502 participants who answered this question selected options 1, 2, or 3, just over a third (35%) selected options 4, 5, or 6, and the remaining 10% selected one of options 7 through 10. Recall that this dimension corresponds to the liberal-conservative dimension measured in the US studies.

### Results

#### Verifying Assumptions

Similar to study 1, we verified the assumptions we made based on previous research, and similar to Study 1, all three assumptions were verified. The first assumption, that believers are more politically conservative than non-believers, was confirmed by the results of a *t*-test in which belief in God was the independent variable and political orientation was the outcome, *t*(500) = 6.35, *p* < 0.0001. Note that 502 participants answered the political orientation question. As expected, believers were more right wing (*M* = 4.16) than non-believers (*M* = 3.06). The second assumption, that believers were less likely to believe that climate change is human caused, was confirmed by the results of chi-squared analysis that crossed belief in God with denialism, *χ*^2^(1) = 14.6, *p* < 0.0001. Believers were more likely to deny that climate change was human caused (29.5%) than non-believers were (16%). The third assumption, that people who deny the truth about climate change would be more conservative than those how accepted the truth about climate change, was confirmed by the results of a *t*-test in which denialism was the independent variable and political orientation was the outcome, *t*(500) = 6.67, *p* < 0.0001. Climate change deniers were more right wing, *M* = 4.69, than accepters of the truth, *M* = 3.30.

#### Climate Change-Induced Distress and Belief in God

Next, we examined differences in climate change-induced distress as a function of belief in God. These analyses included the 502 participants who answered the belief in God, political orientation, and climate change distress questions.

The first analysis was a one-way ANOVA in which belief in God was the independent variable and the eight measures of climate change-induced distress were the dependent variables. The results of these analyses are summarized in Table 4. These analyses found a significant main effect for belief in God for all outcomes, and as can be seen from the means presented in Table 4, those who believed in God were less distressed by climate change than non-believers or those who were uncertain.

**Table 4:** Study 2: Climate change-induced distress as a function of belief in God

Outcome	Unadjusted means			Means with political orientation as a covariate			Means with political orientation, sex, and age as covariates		
	Do not believe	Believe	*F*	Do not believe	Believe	*F*	Do not believe	Believe	*F*
Worried about future	4.02	3.71	5.43*	3.95	3.79	1.35	3.94	3.81	.874
Feel anxious climate change	3.88	3.54	6.07**	3.81	3.60	2.16	3.82	3.57	2.96^a^
Afraid effects will be fatal	4.20	3.81	8.61**	4.11	3.90	2.36	4.13	3.91	2.50
Feel dread about consequences	3.75	3.41	5.51*	3.67	3.49	1.56	3.67	3.51	1.08
Feel sad	4.16	3.79	7.43**	4.07	3.87	1.95	4.08	3.87	2.14
Awareness of climate change takes away joy	2.89	2.54	6.75**	2.83	2.58	3.54^a^	2.82	2.59	2.87^a^
Future seems hopeless	3.67	3.17	11.71***	3.59	3.24	5.58*	3.59	3.24	4.99*
Feel sorry about consequences	4.44	4.00	11.24***	4.36	4.10	3.58^a^	4.38	4.09	4.27*

*F* ratio is the effect for belief in God. *p < .05, **p ≤ .01, ***p ≤ .001, ^a^*p* = .06. *n*s between 499 and 502

The next analysis was a one-way ANCOVA in which belief in God was the independent variable, the eight measures of climate change-induced distress were the dependent variables, and political orientation was a covariate. The results of these analyses are summarized in Table 4. As expected, after controlling for political orientation, all differences in distress as a function of belief in God were smaller, and all but one was not significant at *p* < 0.05. Note that two differences remained significant at *p* = 0.06. The adjusted means are presented in Table 4.

The next analyses were ANCOVAs that included sex and age as covariates. Age was measured in self-reported years. Gender was represented with a dummy-coded variable, 0 = male, 1 = female. This was a student sample, and so education was not included as a covariate. The adjusted means are presented in Table 4. Similar to the results of Study 1, as can be seen from the adjusted means in this table, the inclusion of sex and age did not alter the results meaningfully. Differences between the two sets of adjusted means were in the second decimal place.

## Study 3

### Methods: Sample and Measures

Participants in Study 3 were 1154 adults living in Poland who were recruited by a survey company (53% women, *M*_age_ = 46.6; SD = 15.5). Education was measured with a 6-point scale ranging from primary/middle school only to university degree. Just less than half (46.7%) of participants had a post-secondary education or more. Ethnicity was not measured. See Study 2.

Participants answered a question about their belief in God: “Do you believe in God?” using a response scale with options: Definitely not, Not, Probably not, Difficult to say, Probably yes, Yes and Definitely yes. To provide a metric that was similar to that used in the other studies, responses Definitely not and Not were recoded as not believe, responses Probably not, Difficult to say, Probably yes were recoded as uncertain, and responses Yes and Definitely yes were recoded as believe in God. This resulted in 18.5% non-believers, 42% uncertain, and 39.5% believers. This is roughly comparable to the distribution of belief found in Study 1. The study was conducted in December, 2021.

Participants described their beliefs about climate change by responding to the same question used in Studies 1 and 2. The percent endorsing each response were: “I do not think the climate is changing” (4.6%); “It is not certain if the climate is changing” (4.8%); “I think that climate change is happening, but it is entirely caused by natural processes” (8.3%); “I think that climate change is happening, but it is mainly caused by natural processes” (10.1%); “I think that climate change is happening, and it is due to an equal mix of natural processes and human activity” (28.5%); “I think that climate change is happening, and it is mainly caused by human activity” (35.0%); and “I think that climate change is happening, and it is entirely caused by human activity” (8.8%). Similar to studies 1 and 2, a measure of climate change denial was created (endorsing options 1 through 5). In sharp contrast to Studies 1 and 2, 56.2% of participants did not acknowledge the reality of climate change.

Climate change-induced distress was measured with nine items. Each was preceded by the stem “How much do you feel…”, and response options were: (1) I don’t feel like that, (2) To a small extent, (3) Moderately, (4) To a great extent, (5) This is exactly how I feel. The items, summary statistics, and frequency counts for these measures are presented in Table 5. Mean responses tended to be near the scale midpoint (except for the item about joy of life), and the distributions of most of the items were not characterized by any pronounced skew.

**Table 5:** Study 3: Descriptive statistics and of responses for measures of climate change distress. Percent for each response in parentheses

Item			Response				
	*M*	SD	1	2	3	4	5
Due to climate change, I am worried about what the future will be	3.17	1.12	111 (9.6)	181 (15.7)	393 (34.1)	340 (29.5)	129 (11.2)
I am concerned about the progressive climate change	3.10	1.11	122 (10.6)	174 (15.1)	436 (37.8)	309 (26.8)	113 (9.8)
I am afraid that the effects of climate change will be fatal	3.29	1.14	105 (9.1)	147 (12.7)	376 (32.6)	363 (31.5)	163 (14.1)
I feel anxious when I think about the consequences of climate change	3.02	1.14	150 (13.0)	183 (15.9)	420 (36.4)	295 (25.6)	106 (9.2)
I become deeply sad when I think about the progressive climate change	3.06	1.16	145 (12.6)	177 (15.3)	422 (36.6)	285 (24.7)	125 (10.8)
Thinking about climate change makes me feel angry and/or frustrated	2.91	1.11	154 (13.3)	223 (19.3)	428 (37.1)	266 (23.1)	83 (7.2)
Climate change makes the future seem to hopeless to me	2.90	1.13	168 (14.6)	206 (17.9)	432 (37.4)	265 (23.0)	83 (7.2)
The awareness of the consequences of climate change deprives me of the joy of life	2.63	1.12	226 (19.6)	265 (23.0)	430 (37.3)	171 (14.8)	62 (5.4)
I feel regret watching the consequences of climate change	3.20	1.12	115 (10.0)	156 (13.5)	400 (34.7)	348 (30.2)	135 (11.7)

Political orientation was measured with a single item: How do you generally see yourself, more on the side of the “left” or more on the side of the “right wing,” with responses labeled (1) definitely left (7.1%), (2) on the left (11.3%), (3) somewhat left (13%), (4) in between (42.5%), (5) somewhat right (11.3%), (6) on the right (8.1%), and (7) definitely on the right (6.8%).

### Results

#### Verifying Assumptions

Similar to Studies 1 and 2, we verified the assumptions we made based on previous research, and similar to Studies 1 and 2, all three assumptions were verified. The first assumption, that believers are more politically conservative than non-believers, was confirmed by the results of a one-way ANOVA in which belief in God was the independent variable and political orientation was the outcome, *F*(2, 1151) = 80.9, *p* < 0.0001. As expected, believers were more conservative (*M* = 4.48) than both non-believers (*M* = 3.04), and those who were uncertain (*M* = 3.75) (ps < 0.0001 for both comparisons using Scheffe test).

The second assumption, that believers were less likely to recognize that climate change is human caused, was confirmed by the results of chi-squared analysis that crossed belief in God with denialism, *χ*^2^(2) = 31.1, *p* < 0.0001. Non-believers were less likely to deny that climate change was human caused (39.4%) than believers (61.8%) or those who were uncertain (58.4%). It should be noted that the percent of believers who were deniers and the percent of those who were not certain did not differ. The third assumption, that people who did not know the truth about climate change would be more conservative than those how accepted the truth about climate change, was confirmed by the results of a *t*-test in which denialism was the independent variable and political orientation was the outcome, *t*(1152) = 9.06, *p* < 0.0001. Climate change deniers were more right wing, *M* = 4.25, than accepters of the truth, *M* = 3.47.

#### Climate Change-Induced Distress and Belief in God

Next, we examined differences in climate change-induced distress as a function of belief in God. These analyses included the 1153 participants who answered the belief in God, political orientation, and climate change distress questions.

The first analysis was a one-way ANOVA in which belief in God was the independent variable and the eight measures of climate change-induced distress were the dependent variables. The results of these analyses are summarized in Table 6. These analyses found a significant main effect for belief in God for all outcomes except for worry about the future. Pairwise comparisons found that those who believed in God were less distressed by climate change than non-believers or those who were uncertain, except for worry about the future and taking away joy for which the comparisons were not significant. The means are presented in Table 6.

**Table 6:** Study 3: Climate change-induced distress as a function of belief in God

Outcome	Unadjusted means				Means with political orientation as a covariate				Means with political orientation, sex, age, and education as covariates			
	Do not believe	Not certain	Believe	*F*	Do not believe	Not certain	Believe	*F*	Do not believe	Not certain	Believe	*F*
Worried about future	3.24	3.20	3.10	1.67	3.09	3.18	3.20	< 1	3.12	3.18	3.19	< 1
Concerned about progressive climate change	3.27	3.11	3.01	4.03*	3.15	3.09	3.10	< 1	3.17	3.09	3.09	< 1
Afraid effects will be fatal	3.45	3.28	3.22	3.04*	3.30	3.25	3.32	< 1	3.33	3.26	3.30	< 1
Feel anxious about consequences	3.14	3.07	2.91	3.59*	3.01	3.05	3.00	< 1	3.04	3.04	2.99	< 1
Deeply sad when think about progressive climate change	3.18	3.10	2.96	3.10*	3.04	3.08	3.05	< 1	3.08	3.07	3.03	< 1
Thinking about climate change feel angry or frustrated	3.00	2.96	2.82	2.69a	2.88	2.94	2.90	< 1	2.91	2.94	2.89	< 1
Future seems hopeless	3.03	2.97	2.77	5.22**	2.91	2.95	2.85	< 1	2.93	2.95	2.52	< 1
Awareness of climate change takes away joy	2.59	2.73	2.55	3.17*	2.51	2.72	2.61	2.82^a^	2.52	2.71	2.61	2.29
Regret consequences	3.37	3.21	3.11	3.85*	3.23	3.19	3.20	< 1	3.26	3.19	3.19	< 1

*F* ratio is the effect for belief in God. *p < .05, **p ≤ .01, ^a^*p* = .06. *n* = 1154 for all analyses

The next analysis was a one-way ANCOVA in which belief in God was the independent variable, the eight measures of climate change-induced distress were the dependent variables, and political orientation was a covariate. The results of these analyses are summarized in Table 6. As expected, after controlling for political orientation, all differences in distress as a function of belief in God were rendered not significant. Note that the main effect for belief in God was significant at 0.06 for taking away joy. The adjusted means are presented in Table 6.

The next analyses were ANCOVAs that included sex, age, and education as covariates. Age was measured in self-reported years. Gender was represented with a dummy-coded variable, 0 = male, 1 = female. Education was represented using the 6-point scale mentioned previously. The adjusted means are presented in Table 6. Similar to the results of Studies 1and 2, as can be seen from the adjusted means in this table, the inclusion of sex, age, and education did not alter the results meaningfully. Differences between the two sets of adjusted means were in the second decimal place.

## Study 4

### Methods: Sample and Measures

Participants in Study 4 were 1098 adults living in the USA who were recruited by a survey company (72% women, *M*_age_ = 50.4; SD = 16.2).^1^ Education was measured with a 7-point scale ranging from did not graduate high school to doctoral degree. Just over half the sample (50.4%) had a BA or more. Ethnicity was not measured. Participants answered a question about their belief in God using the same question used in Study 1: “Do you believe in God?” with a response scale of “no,” “not certain,” and “yes.” Of the 1069 participants who answered this question, 149 (13.9%) answered no, 131 (12.3%) answered not certain, and 789 (73.8%) answered yes. The study was conducted in May of 2022.

Participants described their beliefs about climate change by responding to the same question used in the previous three studies. The percent endorsing each response were: “I do not think the climate is changing” (3.5%); “It is not certain if the climate is changing” (2.4%); “I think that climate change is happening, but it is entirely caused by natural processes” (6.2%); “I think that climate change is happening, but it is mainly caused by natural processes” (8.7%); “I think that climate change is happening, and it is due to an equal mix of natural processes and human activity” (29.1%); “I think that climate change is happening, and it is mainly caused by human activity” (32.6%); and “I think that climate change is happening, and it is entirely caused by human activity” (17.4%). Similar to the previous studies, a measure of climate change denial was created (endorsing options 1 through 5). In sharp contrast to Studies 1 and 2, but comparable to Study 3, 51% of participants did not acknowledge the reality of climate change.

Climate change-induced distress was measured with two items, and the response scales were the same as those used in Study 1, i.e., 5-point scales labeled: (1) I do not feel this way at all; (2) I feel this way slightly; (3) I feel this way moderately; (4) I feel this way strongly; and (5) This is exactly how I feel. The items, summary statistics, and frequency counts for these responses are presented in Table 7. The mean responses to these two items were close to the midpoint of the scale, and the distributions of responses to both items are not strongly skewed.

**Table 7:** Study 4: Descriptive statistics and of responses for measures of climate change distress. Percent for each response in parentheses

Item			Response				
	*M*	SD	1	2	3	4	5
I worry about what the future will be like because of climate change	3.22	1.47	201 (18.6)	176 (16.3)	183 (16.9)	225 (20.8)	297 (27.4)
Climate change makes me feel anxious	2.89	1.47	273 (25.3)	189 (7.5)	212 (19.7)	186 (17.3)	217 (20.1)

Political orientation was measured using the same item used in Study 1: Please describe your general political orientation using the following scale (with percent endorsing each option of the 1034 participants who answered this question): (1) Very liberal (16.3%), (2) Liberal (17.8%), (3) Slightly liberal (8.7%), (4) Moderate, middle of the road (29.6%). (5) Slightly conservative (6.6%), (6) Conservative (10.7%), and (7) Very conservative (10.3%).

### Results

#### Verifying Assumptions

Similar to the previous studies, we verified the assumptions we made based on previous research, and similar to the previous studies, all three assumptions were verified. The first assumption, that believers are more politically conservative than non-believers, was confirmed by the results of a one-way ANOVA in which belief in God was the independent variable and political orientation was the outcome, *F*(2, 1007) = 65.5, *p* < 0.0001. As expected, believers were more conservative (*M* = 4.04) than both non-believers (*M* = 2.31), and those who were uncertain (*M* = 3.08) (ps < 0.0001 for both comparisons using Scheffe test). The second assumption, that believers were less likely to recognize that climate change was human caused, was confirmed by the results of chi-squared analysis that crossed belief in God with denialism, *χ*^2^(2) = 14.4, *p* < 0.0001. Believers were more likely to deny that climate change was human caused (58.3%) than either non-believers (24.8%) or those who were uncertain (40.5%). The third assumption, people who did not know the truth about climate change would be more conservative than those how accepted the truth about climate change was confirmed by the results of a *t*-test in which denialism was the independent variable and political orientation was the outcome, *t*(1032) = 14.36, *p* < 0.0001. Climate change deniers were more conservative, *M* = 4.42, than accepters of the truth, *M* = 2.87.

#### Climate Change-Induced Distress and Belief in God

Next, we examined differences in climate change-induced distress as a function of belief in God. These analyses included the 998 participants who answered the belief in God, political orientation, and climate change distress questions.

The first analysis was a one-way ANOVA in which belief in God was the independent variable and the two measures of climate change-induced distress were the dependent variables. The results of these analyses are summarized in Table 8. These analyses found a significant main effect for belief in God for both outcomes. Comparisons of means for believers versus non-believers and uncertains were both significant at *p* < 0.001. As can be seen from the means presented in Table 8, those who believed in God were less distressed by climate change than non-believers or those who were uncertain.

**Table 8:** Study 4: Climate change-induced distress as a function of belief in God

Outcome	Unadjusted means				Means with political orientation as a covariate				Means with political orientation, sex, age, and education as covariates			
	Do not believe	Not certain	Believe	*F*	Do not believe	Not certain	Believe	*F*	Do not believe	Not certain	Believe	*F*
Worry about future	3.79	3.56	3.08	17.83	3.31	3.36	3.21	< 1	3.32	3.35	3.21	< 1
Feel anxious	3.30	3.10	2.80	8.14	2.81	2.90	2.93	< 1	2.81	2.87	2.94	< 1

*F* ratio is the effect for belief in God. All effects for unadjusted means were significant at *p* < .0001. For unadjusted means, all ps of pairwise comparisons (Scheffe test) significant at .01 or less except for believers versus not certain in the analysis of feeling anxious. *n*s = 998 and 995 for the first and second measures

The next analysis was a one-way ANCOVA in which belief in God was the independent variable, the eight measures of climate change-induced distress were the dependent variables, and political orientation was a covariate. The results of these analyses are summarized in Table 8. As expected, after controlling for political orientation, all differences in distress as a function of belief in God were rendered not significant. The adjusted means are presented in Table 8.

The next analyses were ANCOVAs that included sex, age, and education as covariates. Age was measured in self-reported years. Gender was represented with a dummy-coded variable, 0 = male, 1 = female. Education was represented using the 7-point scale mentioned previously. The adjusted means are presented in Table 8. Similar to the results of Studies 1, 2, and 3, as can be seen from the adjusted means in this table, the inclusion of sex, age, and education did not alter the results meaningfully. Differences between the two sets of adjusted means were in the second decimal place.

## Discussion

Across four samples of students and adults in two countries, we found that believers in God were less distressed by climate change than non-believers, and that such differences either disappeared or were reduced when individual differences in left–right political orientation (or liberal-conservative in the USA) were taken into account. These results occurred regardless of how these three constructs were measured, including various measures of distress.

### Use of Belief in God as a Measure of Religiosity

The present study used a straightforward and simple measure of religiosity, belief in God. We used belief in God because we were not certain which of the myriad measures of religiosity would be most appropriate for present purposes, and belief in God is applicable to various faiths, denominations, and sects. In three of four studies, participants were given three options, belief, non-belief, and uncertain. By and large, the results suggested that those who were uncertain were more similar to non-believers than they were to believers.

By design, our measure of belief in God did not include intensity of belief, how important religious beliefs were to people, how involved people were with their faith (e.g., attendance at religious functions), and various other aspects of religiosity. Nevertheless, as discussed by Arbuckle and Konsiky (2015), at least for Christians in the USA, concerns about the environment are negatively related to other measures of religiosity such as personal importance of religion and attending functions. The extent to which this characterizes other faiths in other countries remains to be seen.

Although we have no reason to suspect this, it is possible that if we had examined relationships between climate change distress and other measures of religiosity, we would not have found that political orientation accounted for differences in climate change distress as a function of religious belief. For example, attachment to God has been proposed as a way for people to deal with stress (Kirkpatrick, 2005), and some have argued that religious faith serves as way of dealing with anxiety about mortality (Terror Management Theory; Greenberg et al., 2020). It is an open question if political orientation could account for differences in climate change distress as a function of these conceptualizations of religiosity if such differences existed.

We also did not measure the extent to which people thought that God would somehow rescue or save humanity from climate change, or that the “end times” are approaching (Jones et al., 2014), or that the “world is disposable” (Braterman, 2020), views popular among some Christians in the USA, particularly evangelicals. Clearly, for people who believe that God will fix things, or that it does matter if humanity destroys the natural world, or it’s all going to end soon, climate change is simply unimportant. See Ecklund and Scheitle (2018, pp. 106–107) for a discussion of this type of thinking.

### Relationships Between Belief in Science and Belief in God and Political Orientation

It is abundantly clear that the credibility of science is under attack, primarily by right-wing politicians and organizations funded by corporations and wealthy individuals with right-wing political orientations (e.g., Lewandowsky et al., 2022). Although Lewandowsky et al. focused on science denialism in terms of the COVID-19 pandemic, they also explained that the same types of attacks (misinformation, false experts, etc.) were occurring in other areas such as climate change. Along the same lines, Dunlap and Jacques (2013) describe how conservative think tanks fund the publication of books that deny the fact that climate change is human caused.

These attacks have had an effect. For example, analyses of the US General Social Survey found that public trust in science became increasingly polarized from 1978 to 2018 (Li & Qian, 2022). Moreover, although much attention has focused on such relationships as they exist in the USA, this is an international phenomenon.

Broadly speaking, the available research suggests that religiosity is negatively related to confidence in science (e.g., World Values Survey, 2010–2014; Chan, 2018). Ecklund and Scheitle (2018) suggest that this is because people of faith may tend to believe more than non-believers, that science, particularly climate change science, is politically motivated. As astonishing as this may be given the clear involvement of the political right in promoting climate change denial, the belief that climate change science is a project of the political left is a reality for many, and people of faith appear to be over-represented in this group.

### Implications of the Present Results for Health and Well-Being

Although at first glance, it might seem that lower climate change distress is healthier than higher climate change distress, such a conclusion is predicated on the false assumption that distress is bad per se. As discussed by Maloney et al. (2011), and as demonstrated by Cypryańska and Nezlek (2020), Verplanken et al. (2020), and Verplanken and Roy (2013), fear and anxiety can serve as a motivator for adaptive behaviors. See also Brosch (2021). In the case of climate change, distress is positively related to engaging in climate change mitigation behavior and supporting pro-environmental policies, both of which are health promoting.

Admittedly, distress is not pleasant or enjoyable, but distress can serve as a warning sign that something is wrong and needs to be changed. For example, as depicted in the popular film “Don’t Look Up” in which a politically conservative government discourages people from accepting the reality of an approaching comet that will destroy the Earth (McKay, 2021), ignoring distress and the implications of climate change will have cataclysmic and irreversible consequences. So, the relative lack of climate-induced distress among believers versus non-believers, which are supported by right-wing political beliefs, is not health promoting for anyone.

### Limitations and Future Directions

The present studies were conducted in Poland and the USA, two countries in which conservative (right wing) politicians presently hold considerable power, and in which there are prominent conservative religious leaders. When these studies were conducted, the ruling party in Poland (Peace and Justice) advocated many of the same right-wing policies advocated by the increasingly conservative Republican party in the USA, including opposition to policies that are needed to fight climate change. The views about climate change (i.e., denial of the sources or seriousness of climate change) of Father Tadeusz Rydzyk, a prominent cleric in Poland (who oversees national radio and television networks), are not very different from the views of many prominent (often evangelical) preachers in the USA as well as many conservative Catholic clergy in the USA. Although we believe that the present results make theoretical sense in terms of individual-level processes, it may be that the relationships we found occur only when there is a combination of conservative politicians and conservative religious leaders.

The effect sizes we found were not large. Although the main effects for belief in God varied somewhat across studies and measures, no main effect accounted for more than 10% of the variance in any outcome, and some main effects accounted for only 1–3% of the variance. This reflects the fact that climate change-induced distress is influenced by multiple factors of which belief in God is only one. Nevertheless, as discussed by Ellis (2010, pp. 35–38), small effects can be meaningful when they accumulate and differences between outcomes depend upon small differences in circumstances, for example, in an election that is decided by less than 1% of the total votes. As organizers of “Get out the Vote” programs note, every vote matters.

## Conclusions

Contrary to those who argue that there is something inherent in religious belief that predisposes people to deny or ignore climate change, the present results suggest that it is the (growing) confluence of faith and conservative political orientation that is responsible for the fact that some people of faith tend to deny climate change or actively oppose efforts to combat it. Once we controlled for political orientation, there were no differences between believers and non-believers in the distress they felt about climate change. Moreover, at a broader level, this lack of a difference in distress can be interpreted as a lack of a difference in concern, which is positively related to the likelihood people will engage in climate change mitigation behavior.

In some senses, we consider the present paper to be “proof of concept.” The samples in Studies 1 and 2 were convenience samples of collegians. Regardless, given that the results of Studies 1 and 2 were very similar to the results of Studies 3 and 4 (using nationally representative samples), whatever differences exist between collegians and the general population did seem to play a role in the relationships we examined. Although the national samples we collected were representative of Poland and the USA, we cannot conclude that the relationships we found characterized all Poles and all Americans. It is possible that the relationships we found vary across different religious traditions, different conceptualizations of religiosity, and different societies (Arbuckle & Konisky, 2015; Taylor et al., 2020). Regardless, we believe that the present results provide a framework for future research examining how conservative politics combine with religious faith to reduce the distress people feel as a result of climate change. Although the reduction of distress may seem to be desirable, in the case of climate change, climate change distress can lead to climate change action, action for which there is a pressing need.

## Data Availability

The data for all studies are available via the Open Science Foundation repository, https://osf.io/2h6er/?view_only=f636a092bd8c4dd081990d86dbfe9511.

## Footnotes

1 Due to an error specifying the sampling parameters, the sample was not representative in terms of sex. Nevertheless, including sex as a covariate did not change the reported results.
